# Experimental Physiology

**Published:** 1870-08

**Authors:** J. L. Prevost

**Affiliations:** Member of the Biological Society


### Original Translations.

ARTICLE I. — *Experimental Physiology.* Experimental Investigations relative to the Directions of Rotary Movements due to Unilateral Encephalic Lesions. By Dr. J. L. PREVOST, Member of the Biological Society.

OF THE VARIOUS ROTARY MOVEMENTS OBSERVED IN ANIMALS.

Since Magendie directed the attention of physiologists to the phenomena of rotation which are manifested after wounds of one of the middle cerebellar peduncles, numerous experimenters have investigated these phenomena, have described them, and have endeavored to interpret them in various modes.

A consideration of these investigations will convince one that physiologists are far from agreeing upon the varieties of movements induced by lesion of this or that portion of the encephalon.

Before analyzing these different varieties of movements, I will first indicate one cause of the confusion and obscurity which I have observed, and which must have embarrassed all who like myself have been engaged in investigating this subject: I refer to the determination of the direction of the movements induced. I will explain:

The different kinds of circular* movements observed in animals may be arranged in two groups: *the circular movement* (mouvement de mènage)† and the *gyratory* movement or *rotation upon*

* I shall not examine here the movements not included in the circular type, and particularly those which result from wounding the semi-circular canals of the ear as Flourens taught.

† The circular movement (mouvement de mènage) upon a short radius may indeed be compared to the circular movement performed around the

*an axis*, all other movements being only varieties of these two groups. It might even be added that these two groups are themselves but derivatives one from the other, for as Brown-Sequard has remarked:*

"Rolling is, generally, only an exaggeration of turning (or the circular movement), that is to say, when there is diminution of the intensity of the cause which induced the rolling, this same cause induces turning."

It is easy to comprehend the meaning of an observer who, in speaking of the circular movement, says that this movement is made from left to right, or from right to left; the reader, indeed, placing himself in the centre of the circle described by the animal, appreciates perfectly the impression of the observer.

But the same cannot be said, when the rolling movement is considered. When a physiologist, in describing an experiment, says that the animal turns upon its axis from right to left, that it rolls from right to left, that it rolls from the side of the lesion or from opposite to the lesion, the reader is much embarrassed, and may interpret the phenomena in different modes. I am even tempted to believe that discussions have arisen from misunderstandings occurring between experimenters holding essentially the same opinion.

The mechanician or the physicist who describes the rotatory movement of a cylinder, first takes the precaution to describe the position which he holds in relation to this cylinder. If he wishes to describe the direction in which the cylinder turns upon its axis, the physicist would do it by reference to himself, the observer, by supposing himself extended in the axis of the cylinder according to a determinate direction. If the cylinder rolls upon the ground, the physicist would say that the cylinder rolled from one side or another in reference to an observer placed in this or that position.

Physiologists have completely neglected this precaution; hence posterior extremity of the animal, and which has been described by MM. Brown-Sequard and Schiff. See *Compte rendu Soc. Biol.* 1853, p. 167, and *Journ. de Physiolog.*, t. V.; Schiff, *Physiologie des Menschen.* Lahr, 1859, p. 348.

* *Note sur les mouvements rotatoires. Jour. de Phys. du Dr. Brown-Séquard*, 1863, t. III, p. 721.

the obscurity which I have indicated with so much the more propriety, since it appears that the terms *rolling* and *turning* have frequently been employed interchangeably.

Some observers appear to have described the movement substituting themselves in the place of the animal subjected to experiment; others, on the contrary, have described under the name *rolling*, the changes of position of an animal upon the ground, in relation to an observer looking toward the posterior extremity of the animal. This last mode of observation appears to have been most frequently employed, although the plăce occupied by the observer in relation to the animal under experiment is undetermined.

It is thus that M. Vulpian regards the movement of rotation when he makes the remark that the rolling is made in the inverse direction to the circular movement (mouvement de mènage) or turning.*

This difference in the direction of the two movements originates from this cause solely, that in rolling a new factor—the friction of the ground—is added, a point upon which too little stress has been laid, in my opinion. Thus when the experiments are performed upon animals whose mode of progression is swimming, there is often exhibited, as M. Vulpian has observed in the tadpole of the frog,† a movement representing a sort of spiral. What I assert will be better understood if it is remembered that the movements of rotation are made from the side indicated by the direction of the eyes; not that I would make of this deviation the cause of the rotation, an opinion advanced by Henle.

It is sufficient to substitute oneself for the animal under experiment in order to fully appreciate the phenomena. If, indeed, I effect a deviation of my two eyeballs from the right side, I should turn circularly (en mènage) from left to right.

If I am placed upon my feet, I would turn around my axis, always from left to right, in effecting a spinning movement

* Leçons sur le Phys. du Systême Nerveux, p. 586.

† Vulpian mouvement de rotation observés chez les telards de grenouille à la suite des lesions pratiquis sur le centre nerveux,—Examen critique des deverses explication proposés au sujet des mouvements que l'on determine ainsi (Mem. de la Societe de Biologie, 1861.)

(mouvement de toupie).* If I am extended upon the ground I would continue to turn upon my axis from left to right, but encountering the resistance of the ground, I should displace myself in consequence of the friction; and for an observer placed at my feet and observing me rolling, I *should roll from right to left*, whilst continuing to turn myself upon my axis from left to right.

*Rotatory movements produced by lesion of a cerebral hemisphere.*

The rotary movements induced by lesion of one of the cerebral hemispheres, have been much less studied by physiologists than those which depend upon unilateral lesions of the isthmus of the encephalon; this is readily comprehended, for these movements are very distinct only in the higher animals, and are generally less energetic than those which are induced by lateral lesions of the isthmus.

The rotatory movements resulting from lesion of one cerebral hemisphere are all comprised in the class of circular movements (mouvement de mènage) the circle described being greater or less. But is this circular movement executed always in the same direction? Upon this point physiologists are not agreed.

M. Longet,† relying especially upon experiments made upon rabbits, admits, as does M. Lafargue,‡ that in the case of the lesion of the thalamus opticus, rotation is made from the side opposite to the lesion; from the stronger towards the weaker side, as in the case of the lesion of one of the cerebral peduncles. Flourens, on the contrary, by wounding the optic thalamus, found that the rotation was made from the wounded side; whilst a rather deep injury of one of the tubercula bigemina, effected upon a bird, occasioned rotation from the side opposite to the injury, and in reptiles from the side of the injury.

M. Schiff§ sought to explain these variations of authors by

* This expression has been very happily employed by M. Mesnet. See *Physiol. Path. des mouvements circulaires*, by M. Mesnet; *Arch. de Med.*, 1861, t. I.

† Longet, *Traité de Physiologie*, 1850; t. II, p. 408 et 409. See also Physiol. du Systême Nerveux.

‡ Lafargue. Thèse de Paris, 1838.

§ Schiff, *loc. cit.*

showing that the direction of the circular movement varies according to the portion of the optic thalamus which was destroyed. After these experiments upon the higher vertebrata (rabbits), he concludes that the destruction of the anterior three-fourths of the optic thalamus determines circular movements toward the wounded side, whilst destruction of the posterior fourth induces movements towards the side opposite to that of the injury.

M. Brown-Sequard * has subsequently confirmed the opinions expressed by M. Schiff.

The variations of authors have concerned especially the interpretations of injuries which approximate the isthmus; now, in wounding the posterior portion of the optic thalamus, it is possible to involve the peduncles and thus complicate the result. I believe that I can sustain by evidence that at least amongst mammiferæ, the highest in the series, and hence approximating most nearly to man, the circular movement takes place invariably from the side of the injured hemisphere; this opinion has also been admitted by M. Mesnet.†

The proofs which I can furnish are the following:

I shall cite first, four experiments of MM. Philipeaux and Vulpian, which are strongly confirmatory in this direction: "On the 26th of June, lesions of the brain proper were effected upon four dogs, after having removed a disk of the cranium by the aid of the trephine. Upon two of these, a transverse incision of the left cerebral hemisphere was made, care being taken to make the incision as much as possible in front of the corpus-striatum. From a third dog a portion of the left hemisphere, a little more than a cubic centimetre, was extirpated through an opening made in the cranium; and lastly, the left cerebral hemisphere of the fourth animal was *ploughed up* by means of a small iron blade.

"All these dogs, subsequently to the operation, exhibited a slight degree of hemiplegia of the right side, more marked in the anterior than in the posterior paw, and they all turned in circles (en ménage) from right to left, but slowly as they were forced to walk. In cases of this kind the hemiplegia was clearly perceptible although it was slight, for the animals yielded often in the

* Brown-Sequard. *Note sur les mouvements rotatoires.* Journal de Physiologie, 1860, t. III.

† Arch. de Med. *loc. cit.*

limbs of the affected side: these limbs would sometimes even bend under the animal which then fell upon the corresponding side."

These facts furnish valuable data in veterinary medicine. The "sturdy" in sheep furnishes us experiments ready made, in which the deep layers of a hemisphere have been injured, while the superficial are intact.

But veterinarians in their observations do not agree about the direction of the circular movements described by the diseased sheep, and the most opposite opinions have been advanced upon this point.

Attracted by these divergencies of opinion, M. Reynal has prepared an abstract of sixty observations of "sturdy" made with great care, and followed by autopsies; of these he has made a most interesting memoir, in which he compares the direction of the movement executed by the sheep with the position occupied by the entozoon in the brain.

From these numerous facts M. Reynal draws the following conclusions:

"We have observed sixty sheep affected by 'sturdy,' and have taken, or caused to be taken, careful notes of the symptoms and morbid lesions. Upon more than half, we have observed that the sheep turned from the side upon which was the worm, whether this be at a point upon the surface of the brain, or whether it be found in the thickness of the layers which form the superior plane of this organ. We have constantly perceived the animals execute circular movements from the side where the cænurus had established its place of abode. The same was the case when the cænurus was contained in the large ventricles and leaving intact the parts upon which it rested; it impressed no modification upon the cerebral substance other than the production of a thinning of the layers which form the floor of these cavities.

"The sheep turned, on the contrary, the most frequently from the opposite side where the destructive work of the cænurus had involved the deepest layers of the brain, or produced changes in the form of the corpora-striata, the cornua-ammonis, the optic-tha lamior the cerebral triangle. However, when these last lesions exist, the animals do not always turn; often, without following a straight line, they walk forward deviating as much to the right as to the left; locomotion is slow, limited and tottering. It is even usual in this condition to see symptoms of general paralysis, with considerable diminution of sensibility. These last symptoms, somewhat nearly, may be observed, when the cænurus exist in the cerebellum, or when this last undergoes compression, by reason of the space which the worm occupies in the ventricle of the brain. Only there is greater feebleness of movement; they are executed in an automatic manner, the sheep falls frequently, the least obstacle occasioning its fall."

It may be seen, from this passage that the revolution is executed from the side opposite to the seat of the cænurus, only when the deep-seated portions approximating to the encephalic isthmus are found more or less attacked. The results are then very variable, and the same homogeneity in the symptoms is no longer observed.

I shall now present the results with which my personal experiments have supplied me.

The cerebral hemispheres assume, as is well known, a degree of importance, relative to the execution of voluntary movements, so much the greater as the subject is placed higher in the scale of animal life.

The isthmus of the encephalon and its dependencies, on the contrary, acquires, relative to the execution of movements, importance greater in relation to the brain, as it descends the animal scale. This is a well known fact which has been very clearly elucidated by M. Vulpian, in his *Lecons sur le Système Nerveux* (p. 667 et suiv.)

A lesion extending from a cerebral lobe, its removal even will not occasion in the frog any trace of paralysis; in the bird there will be no hemiplegia. In the mammal, on the contrary, hemiplegia will appear, and will become more and more decided in proportion as the subject under consideration may be higher in the mammalian series. Obscure in the rabbit, it will become evident in the sheep, the dog, etc.

But if the hemiplegia produced in the dog, by lesion of a hemisphere, be compared to that which is observed in the same case in man, the difference in the intensity of the symptoms will be striking. In man, a very circumscribed lesion of a cerebral hemisphere, located in the corpus-striatum, for example, will produce hemiplegia of the opposite side, with complete resolution and flaccidity of the limbs. The removal of an entire cerebral hemisphere in the dog will, on the contrary, occasion only a very partial hemiplegia.

There is a fact which must not be forgotten in the examination of rotary movements produced by lesions of the cerebral hemispheres. By making an extensive lesion of a cerebral lobe in a frog, or by the removal of the entire lobe, it is rare to observe them, unless some injury be inflicted on the tubercula bigemina, so voluminous in this animal in comparison with the hemispheres; it is rare, I say, to observe a movement which represents the rotatory movement. I have, however, believed that I have sometimes remarked immediately after the injury, a tendency to leap from the side of the injury, a sort of outline of a rotary movement; but there was in it nothing comparable to the phenomena which are produced when the isthmus is injured in the frog, facts which I shall examine hereafter.

In the mammiferæ, the results are very different. Thanks to the kindness of M. Vulpian, I have been enabled to make in the laboratory of pathological anatomy of the Faculty of Medicine a certain number of experiments upon dogs and rabbits.

These experiments given below, together with the aforesaid facts, demonstrate that lesions of a hemisphere occasion a rotary movement from the side of the injury, and that this movement, better marked in the dog than in the rabbit, probably on account of the predominance of the action of the brain, becomes more manifest when the deep layers of the hemisphere (corpus striatum, optic-thalami, and even the cerebral peduncles) are injured.

In the course of my experiments I have not had the opportunity to verify the opinion of M. Schiff relative to the direction of rotation from the side opposite to the lesion, then from the wounding of the superior portion of the optic thalamus, for I have never produced an injury limited to this level. The rotation produced in my experiments by injury of the optic-thalami has always taken place from the side of the injury. I will say the same of the lesion of the cerebral peduncle in experiment I.

I place at the head of these experiments the observation upon a dog which I presented to the Biological Society, and which is remarkable in consequence of the recovery of the animal, and of the persistence of the rotary movements. There will be found in this experiment several data important in regard to the theory of movements of rotation.

DEEP WOUND OF THE RIGHT HEMISPHERE, TRAVERSING THE RIGHT CEREBRAL PEDUNCLE; ROTATION OF THE HEAD AND OF THE EYES FROM THE RIGHT SIDE; CIRCULAR MOVEMENT FROM LEFT TO RIGHT, REMARKABLE FOR ITS PERSISTENCE; LEFT HEMIPLEGIA.

*Exp.* I. An adult bitch of medium size. The 24th of December, 1867, I perforated with a gimlet, having a diameter of two or three millimetres, the cranial vault of the right side, and buried the instrument deeply into the brain.

The animal described immediately a circular movement from left to right; he inflected the head slightly to the left side, directing the vertex to that side, so that the left eye should be upon a lower plane than the right; he turned, on the contrary, the muzzle to the side of the right shoulder.

The two eyeballs were both directed to the right, the left iris being carried into the internal palpebral angle, the right iris into the external palpebral angle, the left sclerotic visible, on the contrary, in the external angle, and the right in the internal angle of the palpebral openings. The right pupil was very much dilated, the left contracted; there was nystagmus. In describing the circle, the dog bent himself into an arc with its concavity directed towards the right. A decided left hemiplegia was observed, especially marked in the anterior limb, which often yields and shrinks under the animal. In walking, the dog frequently places his left fore paw upon its dorsal face, then the limb doubles up under him, and he falls on his right side.

Sensibility is diminished in the paw of the left side.

During the following days the dog remains downcast and sick, exhibiting the same symptoms; but he is not slow to recover his appetite and spirits, in brief, to recover completely from the general symptoms due to the operation, as may be very well observed; and it is the state which he then presents that I am about to describe in detail.

The dog is intelligent, and recognizes me very well, and each day when I come, before seeing me, he detects my voice and even my step, and manifests his joy by cries, for I am in the habit of feeding him myself.

The paralysis of the left side has diminished, but is however still manifest, especially in the anterior limb, which yields under the animal, especially when he is roughly pushed. The disturbances of mobility have never been very apparent in the posterior limb.

Sensibility is diminished in the two limbs of the left side, for it is necessary to pinch the left paws much more vigorously than the right to induce the animal to withdraw them, or manifest pain. The head is habitually slightly inflected to the left, the vertex being directed to the left side, the left eye nearer to the earth than the right; the muzzle, on the contrary, is turned to the right side, but there is no stiffness in the neck, the head may be turned as well to the left as to the right. The animal, however, of his own accord, only turns it slightly to the left side, and his body is generally bent into a slight curve, the concavity being directed to the right side.

